# Placebo Effect

**DOI:** 10.1100/tsw.2000.9

**Published:** 2000-10-20

## Abstract

Nature and Science ran completely different news line-ups this week. But their lead stories agreed on one thing: patients matter. Nature led with a story about a group of patients who will share in a patent after giving blood and tissue samples to scientists. Science chose to lead with the controversial World Medical Association decision to recommend restricting the use of placebos in certain clinical trials.

The general assembly of the World Medical Association (WMA) is to endorse a new set of international ethical standards, reported Science. The WMA has revised the 1964 Declaration of Helsinki to restrict use of one of medical science's cherished tools: the placebo. Arguing that administering placebos could be life threatening if sick subjects in clinical trials are taken off medication, the WMA feels that scientists should dispense with the classic double-blind experiment. Instead, they now endorse comparisons with treatments that are already "proven" effective. Patient rights groups were ecstatic with the decision. Scientists were less enthusiastic. *Science* quoted U.S. Food and Drug Administration (FDA) official Robert Temple as saying the declaration's paragraph on placebos "doesn't make sense to me." The recommendation (it is not a law, so no one has to obey it) also contradicts the FDA guidelines.

*Nature* reported in its lead story that a group of medical researchers and patients have agreed to share the patent on a recently isolated gene thought to be responsible for a rare genetic disorder of the connective tissue called pseudo-xanthoma elasticum (PXE). The precedent-setting agreement is the product of a lot of blood, sweat, and tears. Scientists from the University of Hawaii did the sweaty work of finding the gene; PXE International, an advocacy group for PXE sufferers, literally gave the blood (and tissue) samples. Who will shed the tears remains to be seen: A mild dispute between the patients and scientists over how to grant access to the gene had not yet been resolved at press time. The University of Hawaii would like to earn a little payback by charging an access fee for the gene, said *Nature*, but PXE is pushing hard for open licensing so that anyone could do research on the gene free of charge.

In other highlights, *Science* reported that the Bill and Melinda Gates Foundation committed another act of noblesse oblige, donating $210 million to fund a Cambridge equivalent to Oxford's Rhodes Scholarship. In a characteristically altruistic prepared statement, Bill Gates, Sr., CEO of the foundation and father of the Microsoft co-founder, said, "We are hoping that the young people we select will be motivated to use their education to put something back into society for the benefit of a much wider community." Officials at Cambridge are reportedly "deeply happy." No kidding.

*Nature* also reported that the National Science Foundation has allocated $52.5 million to fund multidisciplinary ecological, or "biocomplexity," studies. Although many admitted not knowing the exact definition of biocomplexity, reported *Nature*, ecologists applauded the decision. As former president of the Ecological Society of America Diana Wall says, "This is going to change our culture." But you had better apply now, added *Nature*, the U.S. Congress is already moving to cap the money.

*Science* closed out this week with the announcement that the Nobel Prize in medicine went to...but wait, you already know about that. If you read last week's *Nature*, that is. If not, check out *Science* for their rundown of this year's Nobel Prize winners.
